# A social innovation model for equitable access to quality health services for rural populations: a case from Sumpaz, a rural district of Bogota, Colombia

**DOI:** 10.1186/s12939-022-01619-2

**Published:** 2022-02-14

**Authors:** Martha Milena Bautista Gomez, Lindi van Niekerk

**Affiliations:** 1 Centro Internacional de Entrenamiento e Investigaciones Médicas - Universidad Icesi, Av. La Maria, #19-225 Cali, Colombia; 2 London School of Hygiene and Tropical Medicine, Department of Global Health, Cali, Colombia

**Keywords:** Social innovation, Community participation, Healthcare, Low- and middle-income countries, Inclusiveness health

## Abstract

**Background:**

Despite efforts to extend Universal Health Coverage in Colombia, rural and remote populations still face significant challenges in accessing equitable health services. Social innovation has been growing in Colombia as a creative response to the country’s social problems including access to healthcare. This paper presents the findings of a social innovation case study, which was implemented in the rural area of Sumapaz in  Colombia, with the purpose of holistically addressing the health needs of the local population and enhancing health service access.

**Methods:**

A case study methodology was used to investigate and understand the process by which the *Model of Integral Health Care for Rural Areas* was developed and how the various strategies were defined and implemented. Qualitative methods were used in the data collection and all data was analysed using Farmer et al. staged framework on grassroots social innovation which includes growing the idea; implementing the idea; sustainability and diffusion.

**Results:**

The social innovation model was designed as a co-learning process based on community participation. The model was implemented adopting a holistic health approach and considerate of the conditions of a rural context. As a result of this process, access to quality health services were enhanced for the vulnerable rural community. The model has also provided outcomes that transcend health and contribute to individual and community development in different areas eg. agriculture.

**Conclusion:**

The *Model of Integral Health Care for Rural Areas* is a social innovation in health that demonstrates how Universal Health Coverage can be achieved for vulnerable populations through a series of creative strategies which fill systemic voids in access and co-ordination of care, as well as in addresings upstream environmental factors responsible for ill-health.

## Background

Since the 1980s, Colombia, like other countries in Latin America, has undertaken health system reform as a commitment and action towards the achievement of Universal Health Coverage (UHC), underpinned by principles of equity, solidarity and collective action [1, 2]. According to the UHC index, attainment of financial protection and service coverage in Colombia is among the highest in the region, with an index value of more than 80 [3]. However, despite the progress made in access to both preventative and curative services for the Colombian population as a whole, the poorest population groups and those living in rural regions still have lower rates of health service utilization [4, 5]. The lack of access to basic health services and the sub-optimal quality of health services remain undoubtedly two of the most important factors which continues to promote societal inequality in Colombia, which, as Sen [6, 7] points out, constitutes a barrier to human in development and the quality of life of the population.

Within the Latin American UHC framework, and the Colombian health system, there still is a need for equitable health interventions that can fill the gap in service access and provision to vulnerable, rural and low socio-economic populations. The remote settings in which these population groups reside also carry the historical legacy and entanglement of five decades of Colombian armed conflict which adds a further consideration in terms of their health needs [8, 9]. As Wing [10] has argued, “To transform society in support of more fundamental health promotion, a more democratic and ecological approach to scientific study is necessary”. Thus, to meet the needs of the rural Colombian population, an integrated perspective on health is imperative and should be supported by non-traditional solutions that are informed in an interdisciplinary manner, including health sciences but also social and environmental sciences. Likewise, intersectorality and social participation are required in the implementation of these solutions, through establishing links and networks within and across sectors, for the achievement of health equity [11]. A cross-case review by Allotey et al. [12] further highlights the importance of engaging and soliciting the participation of communities, especially those marginalised and excluded, in achieving UHC.

Social innovation has been growing in Colombia, as in other Latin American countries such as Brazil, Chile and Argentina, as a creative response to the region’s social problems and challenges such as access and quality of healthcare, education and poverty [13]. Social innovation can be defined as an initiative, product, process or program that profoundly changes the basic routines, resource and authority flows or beliefs of any social system [14] Moulaert et al. [15] complements this definition by describing three goals social innovations seek to achieve: to meet human needs, particularly those of the most vulnerable population; to raise participation levels of the marginalized, and to achieve empowerment through greater access to resources and increased social and political capacities for people.

Social innovation is a concept that has been viewed through different paradigmatic lenses depending on factors like theoretical approach or geographic contexts. The instrumental or technocratic paradigm views the goal of social innovations as developing more effective, efficient or sustainable solutions that can help address market failures, reduce public spending, amongst others [16]. The institutional or structural paradigm presents social innovation as institutional change or transformation in complex adaptive systems [17]. Then there’s the democratic paradigm, which according to Moulaert, [15] sees social innovation as a way to meet human needs by focusing on increasing the participation and empowerment of communities, creating more access to resources, and increasing social and political capacities. This paradigm is the one through which the case of the Model of Integral Health Care for Rural Areas, in the rural town of Sumapaz (Bogotá, Colombia) has been viewed and analysed.

Social innovation within the Latin American context has several distinguishing features. Domanski et al. [13] describe, the concept of social innovation within Latin America as firmly grounded in the democratic paradigm, in which community or social participation through a rights-based (social justice) approach is a central feature of social innovation. This has arisen in response to historical paternalistic top-down approaches of social development. The region’s most visible and well-known social innovations have emerged from communities and civil society being placed in the driving seat [18]. Community participation as an engine for social transformation has developed widely in Latin America since the 1970s, based on Paulo Freire’s co-learning proposal and participatory action research developed by Fals Borda [19]. Minkler [20] argues, that these approaches make possible to address complex social problems in health, creating interventions that involve the community and resulting in a co-learning processes. A document review focused on social innovation in health conducted by Castro-Arroyave et al. [21] re-emphasises characteristics of the Latin American social innovation approach as being focused on addressing social inequality through social empowerment, fostering equitable power relations, interculturality, intersectorality and multidisciplinary. Another key feature of social innovation within the Latin American context is the synergies between modern and traditional or ancestral knowledge, recognising the value and contribution of indigenous practices [18].

According to Leask et al. [22], co-creation is understood as the process that results in tailored solutions that center the needs and circumstances of certain individuals and that are developed in collaboration with these individuals. As for co-learning, Waterman & al [23] define this concept as the process by which individuals and researchers share their knowledge, create a new understanding, and form action plans together. In the health field, co-creation and co-learning imply the development of collaborative public health intervention by academics working alongside other stakeholders [22]. This process is regarded as a partnership between patients, the public and health professionals.

In this paper, we present the findings of a social innovation case study developed and implemented in a rural post-conflict region in Colombia with the purpose of comprehensively addressing the health needs of the local population and enhancing health service access [24].. The social innovation - ‘*Model of Integral Health Care for Rural Areas, in the rural town of Sumapaz (Bogotá, Colombia)’* - has been implemented as a primary care initiative by the Subred Sur (the main public health institution of the district) for the past 20 years. The purpose of this article is to examine the development and implementation process of a social innovation in health, and its contribution to enhancing equitable access to healthcare and raising the level social empowerment in the local communities. We identify the creation and design of the model as an inclusive co-learning, interdisciplinary and multi-sectoral process that led to innovative place-based strategies inclusive of environmental factors.

### Description of the social innovation in context

The social innovation model was implemented in the Sumapaz﻿ district, one of the most southern areas within the city of Bogota in Colombia. This rural district is part of the Sumapaz National Natural Park, which is considered the largest paramo (high treeless plateau in tropical South America) in the world. Sumapaz has 2692 inhabitants, most of them traditional farmers, their main economic activity is agricultural work and livestock production [25]. The population of Sumapaz is considered a vulnerable and marginalized population because it has historically experienced inequalities, social exclusion and the violence generated by the Colombian armed conflict. The inhabitants are mostly farmers who have high levels of poverty and isolation. Due to these factors, the Sumapaz population has developed resilience through the creation of social organisations [26].

In terms of healthcare, several systemic challenges were faced. First, there was a lack of care co-ordination between the institutions. Second, there was multiple upstream social determinants of health relating to the environment, including the mountainous geography limiting access to the health centres, vast distances from urban specialist care, and the widespread dispersion of the population. In addition, poor socio-economic conditions and factors associated with agricultural work (e.g agrochemical use and physical labor), were additional contributors to poor health outcomes. The most common presenting disorders included musculoskeletal and joint disorders (osteoarthritis and arthritis); disabilities due to injury, cancer, respiratory diseases; intestinal infectious diseases, and as poor nutrition [25].

The social innovation, called the *Model of Integral Health Care for Rural Areas* was designed and implemented by the District of Bogota’s public health institution (Nazareth Hospital during the period 2001–2015 and since 2016, by the Subred Sur). It operates within the framework of the Colombian health system and that of the Compulsory Health Plan. As will be described in more detail below (Stage 2. Intervention), the model has two main aims: the first is to provide equitable and quality health care, and therefore it seeks to create strategies that reduce barriers to access, while ensuring the affordability of quality health services. These strategies take into consideration the culture, lifestyle, social organization of the community, while also valuing their practical knowledge of rural life. It also gives priority to the eco-environmental approach. Intersectoral collaboration was an important principle that supported strategies such as Health Referral routes, Home Health Education visits to achieve this aim. The second aim is the prevention of the disease through experiential pedagogy and co-learning from the environmental and food security perspective. This aim was supported through strategies such as the development of the Chaquén Agricultural Education Park, Home Health Education Visits and Community Health Networks. The model serves the Sumapaz population through two affiliated level-1 health centres (Primary health and some specialities). The frontline staff from these health centres are actively involved in the day-to-day implementation of the model.

## Methods

Qualitative research was undertaken using a case study methodology to investigate and understand the process by which the *Model of Integral Health Care for Rural Areas* was developed and how the various model strategies were defined and implemented. Key features of case study research are its exploratory and explanatory potential of phenomena occurring in ‘open systems’, where context is not controlled as an external variable but permeates the analysis, and variables interact in changing ways over time [27, 28]. Case studies are widely used in exploring the effectiveness of social and cultural strategies in social innovation research [29].

The case was purposely selected from a social innovations in health database [30] managed by TDR, the Special Programme for Research and Training in Tropical Diseases [31]. The innovations in the database were identified through a public crowdsourcing methodology [32]. As the purpose of this research was to understand how social innovations in health can contribute to the achievement of UHC in the Andean region, this case was selected based on four criteria: being a recognised social innovation; implementation in an Andean country; implementation in a rural setting; focus on extending primary care access or affordability of health services to vulnerable populations.

Qualitative data were collected that included: participant observations, in depth individual interviews and focus group sessions, and a review of all relevant documentation (project documents, academic and lay journal articles). The data collection carried out between March 2019 and February 2020. The first stage included the process of crowdsourcing and selection of the case study, and the secondary data collection. In the second stage, the collection of primary data was done during two field work visits to Sumapaz. The first visit was  from 8 - 21 November 2019 and the second one from 1 - 10 February 2020. A total of 8 individual interviews were conducted with members of the Subred Sur *Implementation team*, lasting approximately 1 h in duration. Two focus group sessions, one with 8 members of the management team of Subred Sur for 2 h in duration, and one with 4 community beneficiaries (Food Security Network participants), for 1 h in duration. The formal interviews were complemented with  informal conversations with the implementation team and the beneficiaries throughout the field visit. Participant observations during field visits further provided a great depth of understanding in terms of the context (territory, culture and health conditions) and the implementation processes of the Model.

Field visits were conducted to two implementation sites, San Juan and Nazareth, which included visits to, the Park Chaqúen and visits to the homes of 3 beneficiaries, which took 4 h each. Additional field activities included observing workshops with elderly community members and womens group, that lasted 4- 5 h each. The objectives of these field activities were to observe the main programs and activities and the organisational dynamics (relationships, behaviour and interactions of people). Observations were recorded in field journals, and these were further supported by photographic records taken during the visit.

Field trips were conducted by a social researcher with expertise in working with rural communities and social innovations in health She  also collected  the data and performed the analysis. Data organisation, transcription and coding was done with the help of by a research assistant. All interview data was recorded, transcribed, and then organised and analysed using N-vivo 12 (QSR international). The first phase of the analysis was focused on gaining a broad understanding of the aspects of the social innovation, using a social innovation framework assessing the following components: health and social context; intervention strategies, components and results; implementation strategies including organisational dimensions [33]. In a second phase of analysis, the emerging codes were analysed according to its temporal elements using Farmer et al. [34] framework to better  understand the development of grassroots social innovations over time, which include: stage 1: growing the idea, stage 2: implementing the idea; stage 3: sustainability and diffusion. By using both these frameworks, a deeper understanding as to the underlying mechanisms of the case were identified. An exploration of these mechanisms supported the generation of generalisable health system lessons of relevance to other similar settings.

The case study protocol previously developed as part of a multi-partner study on social innovation in health was translated and adapted to the Latin American context. It was approved by the CIDEIM (Centro Internacional de Entrenamiento e Investigaciones Médicas) Ethics Committee for Research for Human Subjects.

## Results: co-learning and innovative strategies for achieving the inclusiveness health

### Stage 1: Design of the Social Innovation: co-learning process

The *Model of Integral Health Care for Rural Areas* was created in 2001 by the Hospital de Nazareth, a public health  provider institution, which was subsequently replaced  by the Subred Sur district health institution in 2016. Throughout its existence, the innovation has been funded by funding from Bogotá district government. The implemention team is comprised of an interdisciplinary group of health workers, who used a bottom-up approach to identify the contextual needs of the local population. Subsequently, the components of the model were designed as a co-learning process with the community, integrating different disciplines and implemented in collaboration with other health institutions and sectors.

### Contextual needs analysis: holistic approach to understand community health challenges

The first step in initiating this model was the recognition of the contextual differences influencing urban and rural health provision. In the District of Bogotá, the health services are regulated by district health policy. This policy was designed from an urban-centric perspective and thus did not recognise the unique challenges faced by rural populations. For example, the number of beneficiaries who engage in  health promotion activities, the procedures for convening them, and the methodologies used for education cannot be the same as in the rest of the district. In a rural area such as Sumapaz, gathering 10–15 people is a great achievement which requires enlisting the support of local organisations, promoting the activities through information posters in shops , and adopting a pedagogy based in experiential learning. Providing transportation is an important consideration to enable the local community to attend. As a result of many years of iteration, the model was able to meet the health requirements of the Sumpaz population while creating new appropriate to meet the needs of this rural population.

A community needs identification process was conducted to inform the creation of new strategies. This process was deeply embedded in the logic of people and rural life, and conducted from the perspective of the farmers as social actors who have the capacity to influence and transform his or her own reality. In addition, the experiential knowledge of these farmers regarding the environment, culture and agricultural work was leveraged. As highlighted in the quote below, this bottom-up process was in contrast to the traditional top-down implementation approaches usually adopted:

*“…their needs [communities needs] have been identified with them, and they [The community] have been influential participants in the whole process, we have never managed to impose things that occur to us, but always from the reading of what are those needs that the farmer has ...”* (Interview SI01. Implementation team. November 13, 2019)*.*

This needs identification process began back in 2004, when the Nazareth Hospital started conducting an annual health survey (from 2004 until 2016). This enabled gathering accurate information about the population, considering the changing dynamics of the armed conflict and migration. Over time the identification process evolved to include more forms of data and became *“a mixed, rigorous and interdisciplinary methodological process where the localities of the Subred Sur are analyzed in population, environmental, social and economic terms”* [21]. This data has been useful, not only for the public health office but it has also informed the implementation of other district programs and the development of research. The most recent report published by the Subred Sur (2018): *Analysis of conditions, quality of life, health and disease,* presents a socio-epidemiological and geographical analysis. This analysis combines different methods and techniques for collecting quantitative data (health survey) epidemiological (Individual Service in Delivery Records and National Public Health Surveillance System), qualitative (BIT-PESE Methodology: Population, Environmental, Social and Economic), cartographic and documentary data. This combination of methods, data and knowledge has been very successful for understanding the complexity of social reality, interpreting and prioritizing health needs of the Sumapaz population.

The Subred Sur defined five design principles for the *Model of Integral Health Care for Rural Areas* (see Table 1 below) [25].

**Table 1:** Model design principles and rationale

Model Design Principles	Rationale
Family-focused and relationship-centred	The patient is embedded in his or her family unit, recognising their life-course history and understanding their socio-environmental practices inherent in rural life. The relationship with the patient and the family is considered with the same importance, if not greater, than the disease conditions and a means to enhancing the quality-of-care experience.
Political subjects	Patients are regarded as competent social actors with existing capacities, capabilities and knowledge, while identifying opportunities to enhance their personal and family resources.
Health system co-ordination and collaboration	Co-ordination and collaboration between various health service and non-health service stakeholders are promoted in order to reduce administrative and geographic barriers, enhance access to health and enhance appropriateness of services based on need.
Preventative and curative care	Continuity in primary, secondary and tertiary care through integral health routes, while also focusing on preventative care.
Intercultural and Integrated Care	A holistic perspective on health and healing that considers culturally appropriate approaches and with integrated strategies from indigenous and natural medicine.

Source: Authors based on Subred Sur Report (2018) [21]

The contextual needs analysis also assisted the implemention team to identify mutual-learning opportunities, leveraging existing community knowledge while filling gaps or areas where the community lacked knowledge. As a result of the participatory health intervention design process, multiple positive outcomes in health and community development have been achieved.

### Intersectoral, inter-institutional and interdisciplinary cooperation

Building the inter-institutional and intersectoral management capacity were crucial for the successful implementation and sustainability of the model. All relevant institutions came together to deliver a more comprehensive and integrated health model, in so doing strengthening the impact of its action. This process entailed establishing collaborations and networking with different health system actors (health service providers, insurers, local authorities, etc.) These improved linkages within the care continuum, from primary to tertiary specialised care, led to enhanced and timely access to appropriate services for patients and more efficient use of resources by the health system.

*“…that we do not look at health as the levels of care, but in a linear way in sucih a way that from where I identify the need it is addressed ... that that need receives its answer in all its magnitude ... there is the integrity at the moment in which we all unite to provide with the true right to health and to give you a correct and assertive response to your need”* (Interview SI08. Management team. November 14, 2019)*.*

Strategic partnerships were also established with non-health sector actors, such as education, environment and economic development institutions, in order to expand the intervention’s holistic capacity. Cross-sector institutional collaborations  included: the Universidad Nacional, Colciencias, the Botanical Garden, District Environment Office, and the District Economic Development Office. The health care model was designed to incorporate a variety of cross-sectoral expertise, as well as align with the One Health Approach. In a rural and agriculture-based context such as Sumapaz, the One Health Approach recognises ‘the interconnection between people animals, plants and their shared environment’ [35], and the prevention perspective of health, was key to achieving positive impacts in health and wellbeing.

These cross-sectoral collaborations support the delivery of multifaceted training. Training sessions are delivered to the community on environmental issues, and this in turn has resulted in the development of further community social initiatives and community-led agricultural projects. Collaborations with academic institutions support research on various topics such as, the impact of pesticides on children’s learning ability. Both training and research are leading to the creation of new academic and practical knowledge applicable to the rural context of Sumapaz.

Due to its interdisciplinary nature, it was thus imperative for this model to be delivered by a team of diverse professionals. The implementation team, operating from the local health centre as a base, includes: two doctors (one with a specialist qualification in natural and indigenous medicine), a nurse, a dentist, a psychologist, a social worker, health promoters, nursing assistants, occupational and physical therapists. Uniquely, also included in this team are environmental engineers and agronomists, political scientists and anthropologists.

### Stage 2: model intervention strategies

**Fig. 1:**
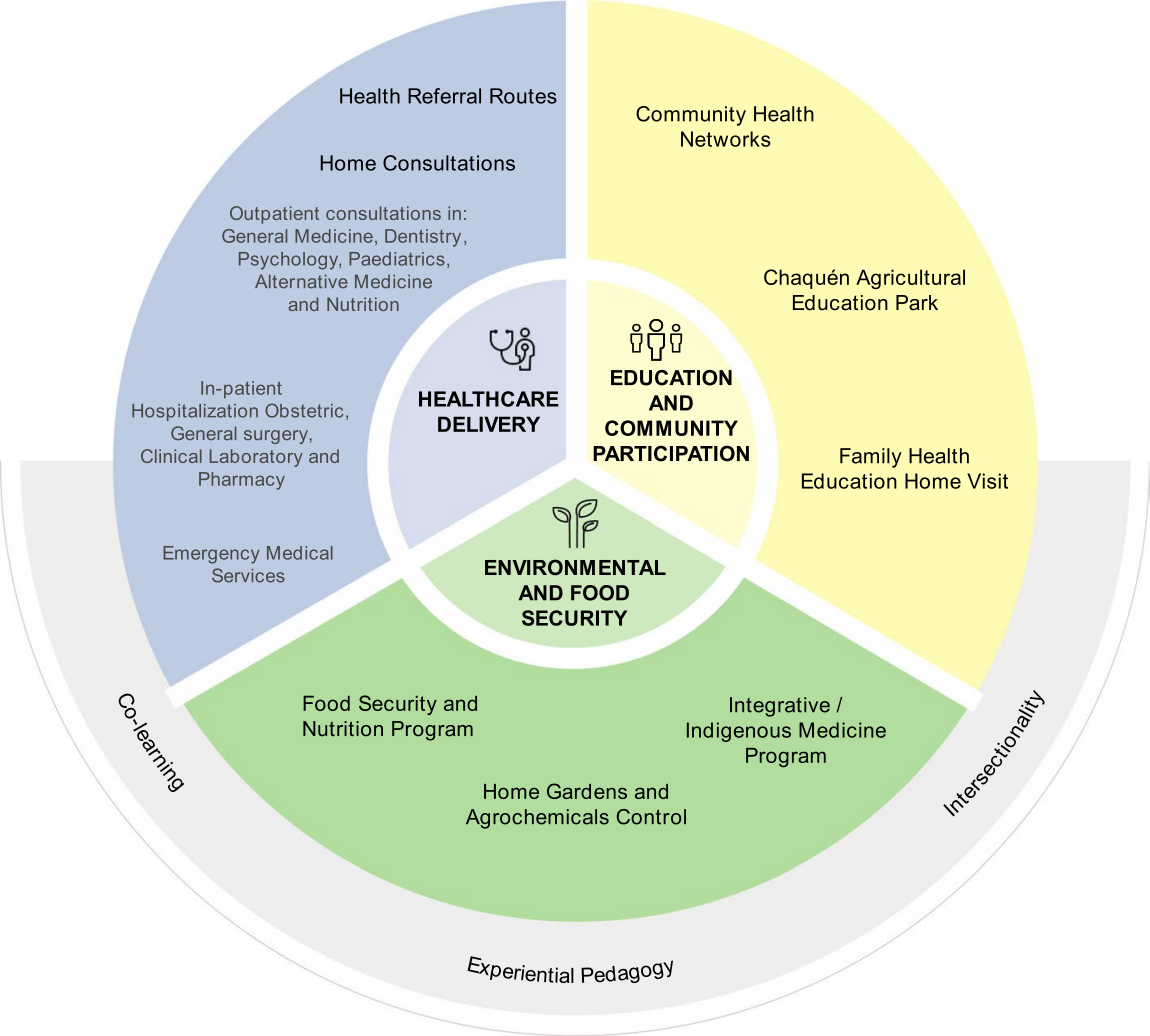
Model of Integral Health Care for Rural Areas. Source: Authors

Based on the contextual need analysis, co-learning and stakeholder collaboration, a set of innovative model strategies emerged, each addressing a specific need surfaced by the local population (Fig. 1).

### Shifting the locus of care: the family home visit

Shifting the locus of care from the health facility to delivering care within the home, was as a strategy adopted to reduce the access barriers associated with long distances to the health facility and difficult mountainous terrain. Instead of health services being concentrated at and accessed only at the health facility, the team of professionals (doctor, nurse, environmental engineers, agronomists and an integrative doctor for the rural sector, among others) would travel to the remote villages to provide care to the family unit (patient and family members) on their farm. During these visits, pre-existing conditions would be monitored, while also screening family members for other health risk factors, diseases, and providing health education.

The home visit experience holds great value for health professionals and the families alike. For the health workers it significantly broadens the health perspective from being only focused on the individual patient, to being the focused on  the whole family. In addition, it provides health workers with a deeper understanding of how the  environmental and territorial context influences upon the health and wellbeing of the community, and thus making it possible to identify risk factors that could lead to ill health. For the family unit, the visit enable them to gain a practical understanding of the relationship between their health and daily lives. The home visits have strengthened and deepened the quality of the relationships between the health team and the families, as expressed by a health worker of the implementation team: *“…the recognition that they [Sumapaz’s community] also make us, so let’s say we [health workers] are one more family”* (Interview SI08. Management team. November 14, 2019).

### Care co-ordination and strengthening linkages to care: health routes

Vast distances, scarce transportation and expensive travel costs are additional barriers in the referral system to urban Bogotá to access specialist health services. To mitigate this problem, ‘health routes’ were adopted. Health routes entail a dedicated support professional tasked with facilitating the patient’s care journey, making and ensuring they receive their referral medical appointments. On a monthly basis, transport is arranged that collect patients at locations in close proximity to their mountainous farms and take them to the urban health centre or hospital as well as return them home again.

This strategy of the health routes was considered of great value by its users, as it contributd simultaneously to patients receiving the appropriate care without delay but also to their adherence to specialist treatments. As mentioned by a member of the implementation team below, this strategy significantly improved service satisfaction for patients:

*“… that the user does not have to suddenly call and that he has his medical appointment in urban Bogotá and that he has to, say, pay the fare from his own means, because here only one bus line passes, then, for an appointment, he would have to go a day before or leave very early, to be able to attend an appointment ..." (*Interview SI04. Implementation team. November 13, 2019)

### Preventing environmental determinants of ill health: an agricultural education centre

One of the most prevalent issues among the Sumapaz population was food insecurity and the accompanying high rates of malnutrition. To address these challenges the Chaquén Park was established in 2007. This interactive agricultural centre was created for the purpose of public health education on agricultural practices, nutrition and environmental determinants of health. The Chaquén Park is an agro-environmental space occupying 2.25 ha of demonstration land. Training is provided to promote home vegetable gardens, organic waste management, environmental restoration by planting trees, the promotion of diets and healthy eating practices, and good agricultural practices such as the reduced use of pesticides.

This educational centre is unique in its approach to education and training. Adopting an experiential pedagogy assisted in building a bridge between health knowledge and ancestral, cultural and contextual knowledge inherent in the rural lifestyle. The use of analogies of rural life supported by practical interdisciplinary activities (see more below) support mutual learning between the health centre staff and the rural community as well as behavioural change. As an example: workshops are often conducted by using approaches such as song and dance, which makes learning both appropriate and accessible to members of the community with lower education levels.

*“…The first activity was a song alluding to farmer life, created by one of the participants and interpreted by the environmental engineer and accompanied by voice and guitar by the doctor of integrative medicine. Then, the agricultural engineer taught them a recipe for rosemary oil for muscle pain, later the integrative doctor showed them a recipe on how to purge with different medicinal plants. Then, the psychologist conducted an activity on memory, peace and reconciliation, which consisted of a simulated river in which the participant had to fish for messages of reconciliation. The workshop ended with lunch cooked by the health team led by the nutritionist, which was a salad made with vegetables from the community garden, and baked cubios [tuber typical from the Andean Region] with bechamel sauce, seeking to try new preparations of this ancestral tuber”* (Field Diary 1. Workshop of the elderly network. November 12, 2019)*.*

For the community, the Chaquén Park is not only a place to receive knowledge and training, but also a space of connection and relationship building with the project team and other health centre staff, as well as with fellow other community members.

*“…From the Chaquén Park I have brought my seeds and have planted them in my gardens with organic fertilizer, and that has helped me a lot for my health and for the health of my family, to have good healthy eating … because for example there we have been taught to make moisturizers, marigold cream, rosemary moisturizer, eucalyptus moisturizer and even [...] taught us how to make preparations with marijuana and alcohol, I even have it at home for pain? ... I like to participate for change from routine at home ...”* (Interview SC05. Community beneficiaries. November 13, 2019).

### Intercultural inclusivity through integrative medicine

The most recent addition to this healthcare model is the Integrative Medicine Program that began in 2016. This innovative strategy is led by a physician specialised in integrative medicine – combining knowledge of conventional, western medicine with that of natural and alternative medicines, as used by indigenous populations. The use of medicinal plants, based on farmers’ ancestral knowledge, as well as alternative therapies are promoted, such as neural therapy for pain [36].

Incorporating an environmental and cultural perspective as part of health delivery was especially important in Sumapaz, where the context is an influencing factor for how disease and wellbeing is viewed by the population. Farmers responded favourably to the use of medicine plants since it is in line with generational and cultural health practices.

*“…We work on the whole topic of medicinal plants, understanding that it is a farmer population that has great adherence [Medication adherence] to these treatments. We liaise with the integrative medicine component and develop actions so that families complement the conventional treatment provided by the Subred Sur and can have greater adherence [Medication adherence] and approach health in a holistic way…”* (Interview S01. Implementation team. November 13 2019).

### Community participation through community education networks

Community networks are another training and education approach adopted by the model. To date, ten networks have been established, each focusing on different health topics: youth, chronic patients, disability, food security, nutritional and environmental safety, women, the elderly, early childhood and healthy habitats. The networks have been the result of a participatory process oriented towards a co-responsibility and co-ownership for good health between community and health institution, as expressed in the following quote: *“…I also believe that to give the recognition that we as health professionals have for the community, is because we recognize them [the community] as a political subject of rights... Then I think that what we have achieved in all these years, is that the community becomes our ally and we allies of them…”* (Interview SI08. Management team. November 14, 2019).

Ongoing training sessions increase participants’ knowledge about their health and their right to health, but also strengthens their capacity as leaders within their community. They are trained to be advocates for the health needs of the most vulnerable groups, developing solutions, and involved in monitoring the work of the Subred Sur health department. As an example: the community network concerning food security, nutrition and environmental safety entailed a three-stage training process taught by the Universidad Nacional de Colombia. In the first stage 40 leaders were trained, 25 of them continued in the second stage, and in the third stage 12 families were given the opportunity to implement their own food security projects with an income generating potential.

*On the other hand, the disability network of San Juan, for them the interest is to learn about leadership, know their policy, know to which entity to make a petition when a person's health access is being violated ... they are not so interested in the theme of let's do practical things…. then each network has its own dynamics...*” (Interview SI01. Implementation team. November 13, 2019).

These networks have also served as effective communication channels, spreading messages in the communities, assisting with the implementation of activities and mobilising behavioural change. The networks have led to improved health knowledge and enhanced community empowerment.

### Stage 3: results - inclusive health and wellbeing

The various strategies of the *Model of Integral Health Care for Rural Areas* were developed iteratively over 20 years. It has brought about significant results: the Sumapaz area has better health outcomes, and the quality and access to health services have improved, as compared to several other similar rural Colombian regions. The outcomes have also transcended health to other social areas, and in so doing created a more widespread social impact.

### Access and healthcare quality

Positive results have been shown in health service coverage with 100% of the rural community having access to health services, through different strategies: medical care in health centres, home visits or community health activities. As is described by one community member:

*"... yes, I have seen that when people feel bad there is a doctor... because here a lady felt bad... and two people from the hospital came to see what was wrong with the lady and to tell her to come down one of these days, they came as if to say, to invite her to the hospital too. Before, you didn't see that, or in comparison to the city you arrive dying and there is nothing, or in the city there is no ambulance to take you home... last year my daughter got a migraine, she fainted, she was at school and they called me to go to school for that reason, and when I arrived they were already taking my daughter to the hospital; that is, they don't wait for the person to get worse, but they start at once and they warn you. ...for me it's a good service..."* (Interview SC05. Community beneficiaries. November 13th, 2019).

Health indicators show positive results related to maternal-perinatal health, as presented below [37–39].

- Maternal mortality ratio at zero during 2016, 2017 and 2018
- Perinatal mortality rate at zero during 2016, 2017 and 2018
- Infant mortality rate at zero during 2016, 2017 and 2018
- Under five years old mortality rate at zero during 2016, 2017 and 2018

The innovation has been recognised multiple times within Colombia and internationally for its quality and achievements. Some of recognitions it achieved includes:

- The Food and Nutritional Security Program had the first place for its contributions to child nutrition awarded by the Éxito Foundation (2003)
- The Chaquén Park was recognised for its contributions to child nutrition awarded by the Colombian Family Welfare Institute (ICBF) (2003), and also as an innovation case for human development by the United Nations Development Programme (UNDP) (2010)
- The care model was recognised as a process of innovation of strategies to achieve that families stop using agrochemicals and can enter organic farming systems by Food and Agriculture Organization (FAO) (2017), and for the environmental contributions they make to Bogotá by The Botanical Garden (2019).

### Impact beyond health: creating value for the community

The long-term investment in education and capacity building have equipped the community with tools and skills that transcend the field of health and contribute to individual and community development in different areas such as education, nutrition, and development of capacities.

The health care model has achieved a holistic improvement on the quality of life of the population through social inclusion mechanisms, empowerment processes, and the inclusion of environmental and food security approaches. As the food security team affirms, in the last four years 20 families have been able to eliminate the use of pesticides in their farming practices, 30 farmers families have adopted organic production systems, and the strategies of good agricultural practices have been adopted by 788 people since the establishment of the Chaquen Park (Subred Sur, unpublished).

Yet, the most important recognition for the implementation team is the value the community places on their work and the fact that the model has been able to institutionalise itself in the territory and the community. One of the most significant influences has been on community leadership. The model has become a school for training leaders as all programs and activities included in this model give theoretical and practical skills in leadership. This approach has been developed over many years and it has built the capacity in the community. When the directors of the Subred Sur Public Health Institution were asked in a focus group why this model has been sustained for 20 years, participants unanimously responded: *“...due to the empowerment of the community*”. The Sumapaz community has become confident in demanding their right to health. Due to their ownership and accountability of this social innovation, they will not allow the quality of health that has been achieved, to be lost.

## Discussion

The *Model of Integral Health Care for Rural Areas* provides an effective approach to enhancing equitable access to quality health services for vulnerable rural communities facing challenges associated with territorial isolation, low socio-economic status and the effects of conflict. Before this primary health model was implemented, the population of Sumapaz had health indicators on par with some of the most isolated regions of Colombia and they were the worst in the Bogota district. After 20 years of sustained implementation and ongoing iteration and refinement, this model is now being replicated to other rural areas of the district.

This model adopts three socially innovative perspectives that reinterprets the conventional views on health service provision: the community as competent agents in their own health and wellbeing; bridging organisational, sectoral and system boundaries and viewing health as medically and environmentally generated.

First, this model’s innovativeness lies in being centred around the person’s and the family’s needs, capacities and resources. As highlighted in the design and development process, the model viewed the local population as not only as passive beneficiaries of care but as knowledgeable and competent agents. As Sen [40] argues, the development of capacities and agency are factors of empowerment that ultimately contribute to widespread social transformation. In the case of the *Model of Integral Health Care for Rural Areas*, strengthening the agency of the community through processeses such as co-creation, co-learning and co-responsibility for health between patients and health institutions, along with an experiential pedagogy adopted to support the development of skills in the farmers, were key factors in generating a transformative change in the health and society of the Sumapaz’s population. The implementing team’s interest was not only to understand the community’s health from an epidemiological perspective but also from their social and environmental reality. The health care model succeeded in social appropriation of knowledge between the communities and the health institution. Given the very accurate understanding of the community’s health needs, strategies could be designed and implement appropriate to the complex rural context. This process of co-learning and social appropriation assisted in developing a person-centred and community-centred model of care. Simultaneously, it increased the social and political capacities of the community [15]. The embedded process of social participation and the involvement of local social organisations and leaders, have been significant in the improvement of community health. These local organisations and leaders are the ones who provide sustainability to the model.

Secondly, this social innovation model demonstrates how an improvement in health indicators can be achieved through bridging organisational, sectoral and system boundaries and creating synergies between stakeholders across health and other sectors. A focus on ‘managing’ the patient’s care pathways across different health system providers made it possible to achieve greater continuity between primary and specialised care. The intersectoral coordination has strengthened the model impact of promotion and prevention efforts. The district health team’s collaboration with other government stakeholders assisted in unlocking budgetary support for the model and it also led to the adjustments of existing health policies, by adapting the urban regulatory framework to be more suitable for the rural context of Sumapaz.

Finally, this model demonstrates the benefits of adopting a multidimensional perspective on health, one inclusive of the environment and indigenous perspectives. Multidisciplinary and intersectorality are key to designing and implementing more effective health services in populations with traditional, indigenous and cultural views on disease and healing. By integrating intercultural perspectives within the model, the uptake and outcome of various interventions were enhanced. The model has successfully incorporated both curative and preventative health strategies, grounded in the One-Health perspective. All the components of the model focusses on enhancing education and empowering people to become health leaders in their communities, and this way encourage the community to change the unhealthy behavioural practices in their home and farms, improving their nutrition and reducing morbidity.

The innovation experienced two major challenges and risks: political changes and financial sustainability. First, the model was created in a favorable socio-political environment with the promotion the primary health care and the recognition of the importance of the rural environment. In some ways the innovation is a symbol of the center-left government that was in power in the district for 16 years. In 2016, when the political orientation of the district changed to center-right, the continuity of the model was at risk. Although some of the health programs were affected by budget cuts, the model was maintained due to the improvement health indicators, the recognition it received, and the community ongoing support for the model. The was model continued under the new political administration by linking up with larger health institutions and services. It has been replicated in other rural areas of the district.

Regarding the financial sustainability, the model is very costly for  several reasons: the private transportation required for both the implementing health team and the community; the higher salaries of rural as compared to urban health personnel, and other contextual factors such as the mountainous terrain and regional conflict requiring security for personal. Its financial sustainability depends on ongoing funding from the district and budgetary cuts would limit its functioning. The model has been successful in diversifying its funding support by also attracting external funding from stakeholders, innovation awards, research studies, and conducting training. However, the mainstay of funding will always depend on the district’s health budget and its willingness to invest in the model.

## Limitations

One of the limitations of the study is the lack of historical quantitative data to establish the impact of innovation, therefore the support of different types of qualitative data was sought. Since the innovator is a public health institution, which means that most workers there specially high ranking figures have strong ties to the political party that it’s in power in the district, some of the opinions and perceptions of participants in the study can have biases associated with political issues, therefore this study sought to include different types of participants who had been involved at different times during the 20 years of implementation.

## Conclusion

The *Model of Integral Health Care for Rural Areas* is a social innovation in health that demonstrates the complexity of the health needs of rural communities affected by a long history of conflict. These challenges can effectively be addressed, not only through new clinical interventions, but through a series of creative strategies which fill systemic voids hindering access and co-ordination of care as well as by addressing upstream environmental factors responsible for ill health. This model was developed over the span of 20-years through a process of community participation, social appropriation of knowledge and collaboration across levels, sectors and disciplines. This model is a is a micro-exemplar of how Universal Health Coverage can be achieved in a person-centred way for vulnerable populations in hard-to-reach areas. All the strategies included in this model has contributed to enhanced access to quality services and it has generated a larger process of social transformation within the community. This paper highlights how a social innovation implemented in a middle-income country context can support the progress towards UHC and people-centred health services.

## Data Availability

The data used for this article is available from the corresponding author on reasonable request.

## Abbreviations

FAO: Food and Agriculture Organization
ICBF: The Colombian Family Welfare Institute
UHC: Universal Health Coverage
UNDP: The United Nations Development Program
TDR: Special Programme for Research and Training in Tropical Diseases
