# Correction to: A social innovation model for equitable access to quality health services for rural populations: a case from Sumapaz, a rural district of Bogota, Colombia

**DOI:** 10.1186/s12939-022-01649-w

**Published:** 2022-04-20

**Authors:** Martha Milena Bautista-Gómez, Lindi van Niekerk

**Affiliations:** 1 Centro Internacional de Entrenamiento e Investigaciones Médicas (CIDEIM), Av. La Maria #19-225, Cali, Colombia; 2 Universidad Icesi, Cl. 18 #122-135, Cali, Colombia; 3 London School of Hygiene and Tropical Medicine, Department of Global Health, Cali, Colombia

**Correction to: Int J Equity Health 21, 23 (2022)**

**https://doi.org/10.1186/s12939-022-01619-2**

After publication of this article [1], the authors reported that the affiliation details for the first author were incorrectly given as '1. Centro Internacional de Entrenamiento e Investigaciones Médicas -Universidad Icesi, Av. La Maria, #19-225 Cali, Colombia' but should have been '1. Centro Internacional de Entrenamiento e Investigaciones Médicas (CIDEIM), Av. La Maria #19-225, Cali, Colombia

2. Universidad Icesi, Cl. 18 #122-135, Cali, Colombia'.

The original article [1] has been updated.
